# Complete Genome Sequence of Nocardiopsis exhalans Strain JCM 11759^T^, Isolated from Indoor Air of a Water-Damaged Private House in Finland

**DOI:** 10.1128/mra.00930-22

**Published:** 2022-11-03

**Authors:** Dan Chen, Ping Mo, Baiyuan Li

**Affiliations:** a Key Laboratory of Comprehensive Utilization of Advantage Plant Resources in Hunan South, College of Chemistry and Bioengineering, Hunan University of Science and Engineering, Yongzhou, Hunan, China; b Hunan Provincial Key Laboratory for Molecular Immunity Technology of Aquatic Animal Diseases, Hunan Provincial Key Laboratory for Health Aquaculture and Product Processing in Dongting Lake Area, Hunan Engineering Research Center of Aquatic Organism Resources and Environmental Ecology, College of Life and Environmental Sciences, Hunan University of Arts and Science, Changde, Hunan, China; University of Southern California

## Abstract

The genus *Nocardiopsis* contains pharmaceutically and biotechnologically important species that produce a wide variety of secondary metabolites with a wide range of biological activities. Here, we report the complete genome sequence of Nocardiopsis exhalans JCM 11759^T^ for a better understanding of its metabolic characteristics and toxin synthesis pathway.

## ANNOUNCEMENT

*Nocardiopsis* species are capable of producing diverse enzymes, compatible solutes, and surfactants that may allow them to prevail in multiple ecosystems (1, 2). Many *Nocardiopsis* species are known to produce a vast variety of bioactive compounds (3). Nocardiopsis exhalans JCM 11759^T^ was isolated from indoor air in a water-damaged private house in Finland in 2001 (4), and the strain name was validated by IJSEM in 2002 (5). This organism may produce toxins that pose a hazard to human health (4), but its genomic properties are still unknown.

The type strain, *N*. *exhalans* JCM 11759 (=DSM 44407 =NBRC 100346 =NRRL B-24123 =VTT E-062617), was purchased from the Japan Collection of Microorganisms (JCM). Cells were streaked onto tryptic soy agar (TSA) plates and incubated at 28°C for 7 days. An individual colony was inoculated into tryptic soy broth (TSB) and incubated at 28°C for 7 days with shaking (220 rpm). Genomic DNA was extracted using a TIANamp bacterial DNA kit (Tiangen, Beijing, China) according to the manufacturer’s protocol. The purity, concentration, and integrity of the DNA sample were checked using a NanoDrop One spectrophotometer (NanoDrop Technologies, Wilmington, DE), Qubit 3.0 fluorometer (Life Technologies, Carlsbad, USA), and 0.35% agarose gel electrophoresis. The DNA samples were simultaneously subjected to the NovaSeq 6000 platform (Illumina Inc., CA, USA) for short-read sequencing and the Nanopore PromethION platform (Oxford Nanopore Technologies, Oxford, UK) for long-read sequencing. Default parameters were used for all software unless otherwise specified. Briefly, the DNA was fragmented and end repaired, and sequencing adapters and barcode labels were added. Then, a short-read sequencing library (paired-end, 150-bp format) was prepared using the NEBNext Ultra II DNA library prep kit (NEB, USA), and a long-read sequencing library with no size selection was prepared using the SQK-LSK109 ligation kit (Oxford Nanopore Technologies). The Illumina library was quantified using a 2100 Bioanalyzer instrument (Agilent Technologies, Santa Clara, CA) and reverse transcriptase quantitative PCR (RT-qPCR). Finally, the libraries were sent to Wuhan Benagen Technology Company Ltd. (Wuhan, China) for sequencing.

The resulting long-read sequences (*n* = 52,523 reads; *N*_50_, 25,024 bp) were assembled, with a coverage depth of 132×, using Flye v2.8.3 (6). The Illumina short reads (*n* = 9,330,736 reads), with a coverage depth of 182×, were used to correct the genome assembly using Pilon v1.22 (7). The Illumina raw sequencing reads were filtered into clean reads using the software SOAPnuke v1.4.0 (8) to discard reads with an N ratio of >10% or more than 50% low-quality bases (Q ≤ 5). Base calling of the Nanopore raw data was accomplished using Guppy v3.1.5, and sequences with a Q score of <7 were discarded. Adapter sequences were trimmed using Porechop v0.2.4 (https://github.com/rrwick/Porechop). The resultant contigs were checked for further joins and circularity using Circlator v1.1.3 (9). The assembly results showed that the two contigs were circular. Sequence comparison was performed using BLAST, and no overlap was found between the two contigs. Using PlasFlow v1.0 software (10), the two contigs were confirmed to be a chromosome and a plasmid. The genomic features were annotated using the NCBI Prokaryotic Genome Annotation Pipeline (PGAP) v6.1 (11). The genome features of *N*. *exhalans* JCM 11759^T^ are summarized in Table 1. The genome comprises 7,597,621 bp; it consists of one circular chromosome (7,506,724 bp) and one circular plasmid (90,897 bp). A total of 6,637 protein coding genes (CDSs), 60 tRNAs, 15 rRNAs, and 3 noncoding RNA genes were detected.

**TABLE 1:** Genome features of *N*. *exhalans* JCM 11759^T^

Name	Length (bp)	GenBank accession no.	GC content (%)	No. of CDSs^*a*^	No. of rRNA operons	No. of tRNA genes
Chromosome	7,506,724	CP099837.1	69.7	6,539	15	60
Plasmid unnamed1	90,897	CP099838.1	69.5	98	0	0

a CDSs, coding DNA sequences.

### Data availability.

The complete genome sequences were deposited at NCBI GenBank under accession numbers CP099837 (genome) and CP099838 (plasmid). The raw reads were deposited in the Sequence Read Archive under the accession numbers SRR21429931 and SRR21429932.
